# TSER polymorphism is not associated with risk of pediatric acute lymphoblastic leukemia: A meta-analysis

**DOI:** 10.1097/MD.0000000000006143

**Published:** 2017-02-17

**Authors:** Zhaohua Qiao, Dan Lou, Li Ruan

**Affiliations:** Department of Pediatrics, The First Affiliated Hospital, and College of Clinical Medicine of Henan University of Science and Technology, Luoyang, China.

**Keywords:** acute lymphoblastic leukemia, meta-analysis, polymorphism, thymidylate synthase

## Abstract

**Background:**

Accumulating studies have explored the effect of thymidylate synthase enhancer region (TSER) variation on risk of pediatric acute lymphoblastic leukemia (ALL) with controversial results. Therefore, this quantitative meta-analysis was performed to assess synthetically the association of TSER variation with susceptibility to develop pediatric ALL.

**Methods:**

The PubMed, ScienceDirect, Google Scholar, Wanfang Database, and China National Knowledge Infrastructure were systematically retrieved to obtain the published case-control studies about the relationship between TSER variation and pediatric ALL risk. The quality assessment of the included studies was preformed and relevant information was collected. Odds ratios (ORs) and 95% confidence intervals (CIs) were applied to evaluate the strength of association.

**Results:**

This meta-analysis finally included 2681 children with ALL and 3854 matched controls from 11 investigations. The quantitative synthesis results found no significant association between TSER variation and susceptibility to pediatric ALL in overall comparisons under 5 genetic models (2R/3R vs 3R/3R: OR = 0.95, 95% CI = 0.84–1.07, *P* = 0.41; 2R/2R vs 3R/3R: OR = 0.99, 95% CI = 0.84–1.16, *P* = 0.90; 2R2R vs 3R/3R+2R/3R: OR = 1.05, 95% CI = 0.92–1.21, *P* = 0.45; 2R/3R+2R/2R vs 3R/3R: OR = 0.97, 95% CI = 0.87–1.09, *P* = 0.63; 2R vs 3R: OR = 1.03, 95% CI = 0.92–1.15, *P* = 0.61). Similarly, there was no significant association existed in the stratification analyses according to ethnicity, control source, and quality score.

**Conclusion:**

This meta-analysis shows that TSER variation is not related to the development risk of pediatric ALL.

## Abstract 2

Supplemental Digital Content is available in the text

## 1 Introduction

Pediatric acute lymphoblastic leukemia (ALL) accounts for 30% of all malignancy diagnosed in children and 80% of pediatric leukemia.^[1]^ Although the clinical outcomes with contemporary treatment regimens of this disease have been well improved, the etiology and precise mechanisms of ALL development have not been fully clarified.^[2–4]^ In general, the interactions between environmental exposures and inherited susceptibility are considered to implicate in the pathogenesis of ALL. Folate metabolism not only supplies the methyl group for proper DNA biosynthesis, it also provides the universal methyl donor for DNA methylation (Supplemental Figure). Plenty of studies have clarified that low folate intake causes uracil misincorporation in the process of DNA replication reactions, resulting in DNA double-strand breakage, chromosomal deletion, and catastrophic DNA repair.^[5,6]^ What is more, hypomethylation of DNA may also cause the activation of proto-oncogenes.^[7,8]^ Emerging evidence has shown that variations in genes encoding folate-metabolizing enzymes disturb the balance of folate metabolism and have been associated with an altered susceptibility to cancer.^[9–11]^

Thymidylate synthase (TYMS) catalyzes the methylation of deoxyuridine monophosphate (dUMP) to deoxythymidine monophospate (dTMP), and maintains the balance of deoxynucleotide pool, which is needed for normal DNA replication and damage repair.^[12,13]^ Therefore, TYMS functions as an essential regulator in the process of DNA biosynthesis, repair, and methylation. The *TYMS* gene with 7 exons locates at 18p11.32. There are several functionally important variants in the *TYMS* untranslated regions, of which thymidylate synthase enhancer region (TSER) variation has been most widely investigated.^[14–16]^ TSER, a tandem-repeat polymorphism, which includes double (2R) or triple (3R) repeats of a 28 bp sequence in the *TYMS* 5′-untranslated enhanced region, may be associated with an alteration in *TYMS* mRNA expression.^[17,18]^ Considering the pivotal role of folate in the development of cancer and the potential influence of TSER polymorphism in the *TYMS* gene on DNA biosynthesis and methylation, it is reasonable that TSER variation might be related to susceptibility to develop malignancies. Increasing studies have found that TSER polymorphism has been linked to human various cancer risks, such as non-Hodgkin lymphoma, breast cancer, and colorectal cancer.^[19–21]^ Recently, numberous investigations have explored the effect of TSER variation on development risk of pediatric ALL, yet the reported results remain controversial. The discrepancies among these studies may be ascribed to the genetic backgrounds difference and relatively small sample size in individual investigation. Therefore, a quantitative meta-analysis was performed to evaluate synthetically the association of TSER variation with pediatric ALL risk.

## 2 Materials and methods

### 2.1 Studies identification

The PubMed, ScienceDirect, Google Scholar, Wanfang Databases, and China National Knowledge Infrastructure were systematically searched to screen reports about the association of TSER variation and risk of pediatric ALL utilizing the following keywords: “childhood” or “pediatric” or “children,” “leukemia” or “acute lymphoblastic leukemia” or “ALL,” “thymidylate synthase” or “TS” or “TYMS,” “polymorphism” or “mutation” or “variation” or “variant.” The latest literature search was performed on January 20, 2016 and there was no language restriction. In addition, the reference lists in the retrieved articles were screened to identify relevant investigations. Ethical approval was not necessary because this study was a meta-analysis.

### 2.2 Inclusion criteria

The following inclusion criteria were applied for literature selection: case-control designed study; confirmed diagnosis for the case group of pediatric ALL; available genotypes distribution data for cases and controls. The letters, case reports, commentary, and review articles were excluded. If the same or overlapping data was reported by multiple articles, we chose the one with larger sample size.

### 2.3 Quality assessment

Two authors independently preformed the quality assessment of included studies according to the Newcastle–Ottawa Scale (NOS).^[22]^ The NOS method, with a maximum score of nine points, includes 3 quality categories: selection, comparability, and exposure evaluation. Studies with more than 6 scores were identified as high quality. Any disagreement was resolved by reevaluation of the originally included studies.

### 2.4 Data collection

The information was collected from each eligible investigation independently by 2 authors: first author's name, publication year, country, ethnicity, sample size, control source, method used for genotyping, genotypes distribution data of the TSER variation in case and control group.

### 2.5 Statistical analysis

The *χ*^*2*^ test was employed to check Hardy–Weinberg equilibrium (HWE) of genotypes distribution frequencies in control groups and *P* < 0.05 was considered as departure from equilibrium. The strength of association between TSER variation and pediatric ALL risk was measured by odds ratios (ORs) and 95% confidence intervals (CIs) under the homozygote model (2R/2R vs 3R/3R), heterozygote model (2R/3R vs 3R/3R), dominant model (2R/3R+2R/2R vs 3R/3R), recessive model (2R2R vs 3R/3R+2R/3R), and allele model (2R vs 3R), respectively. The *χ*^*2*^-test-based *Q* test was performed to estimate the heterogeneity between included studies. When *P* > 0.05, showing that no statistically significant heterogeneity existed, the fixed-effects model (Mantel–Haenszel) was employed to compute the pooled ORs; alternatively, the random-effects model (DerSimonian–Laird) was used. Stratification analyses were carried out based on ethnicity, control source, and NOS score. Sensitivity analysis was conducted by omission of studies deviated from HWE to assess the stability of combined results. Both qualitative funnel plot and quantitative Egger test were employed to assess publication bias. All the statistical tests were done with RevMan v5.3 (The Cochrane Collaboration, Oxford, UK) and STATA v12.0 (Stata Corporation, College Station, TX), and *P* < 0.05 was deemed to have statistical significance.

## 3 Results

### 3.1 Features of included studies

**Figure 1:**
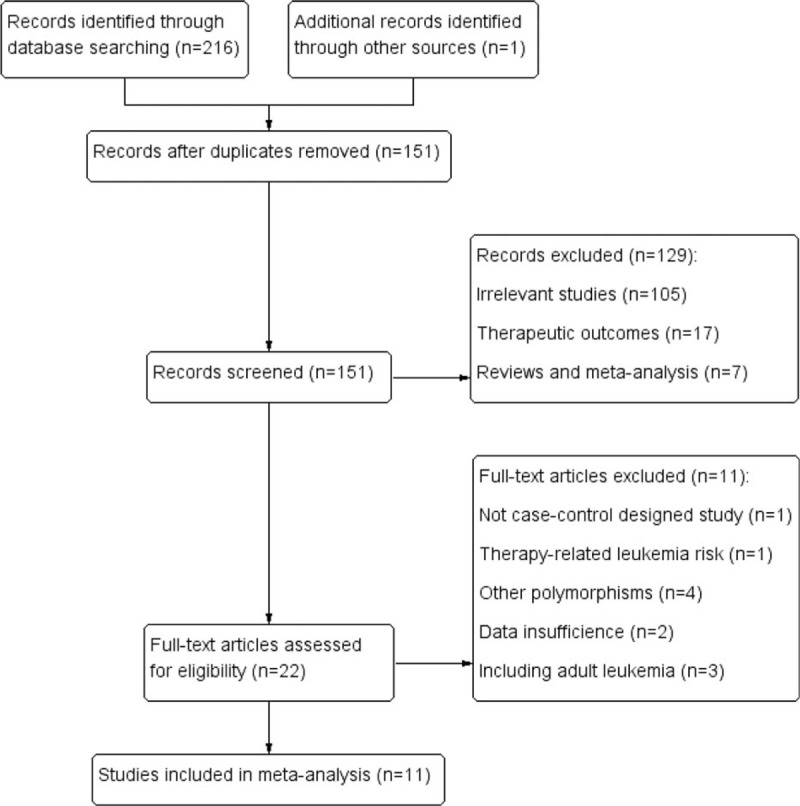
Flow diagram of literature selection process.

Figure 1 shows the flow diagram of the literature selection. Two hundred seventeen relevant records were retrieved based on systematical search. One hundred twenty-nine irrelevant studies and reviews were excluded after glancing the titles and abstracts; during the further assessment, 11 full-text articles were excluded. Finally, this meta-analysis included 2681 children with ALL and 3854 matched controls from 11 studies.^[23–33]^Table 1 lists the main features of eligible investigations. The included cases had a definitive diagnosis according to the universal diagnosis criteria of pediatric ALL. Of these eligible studies, 5 studies were focused on Caucasian descents,^[25–29]^ 4 studies on Asians,^[30–33]^ and 2 investigations on mixed population.^[23,24]^ Four investigations were hospital-based ^[23,30,31,33]^ and 7 were population-based ^[24–29,32]^ designed when classified according to the control source. Four studies were divided into low quality with a NOS score of 4 or 5 points, and 7 with score 6 or greater were assigned as high quality. The alleles and genotypes distribution data of TSER variation in case group and control group are summarized in Table 2. The genotypes distribution frequencies among the controls were in agreement with HWE for all included articles except for 2 investigations.^[23,29]^

**Table 1:**
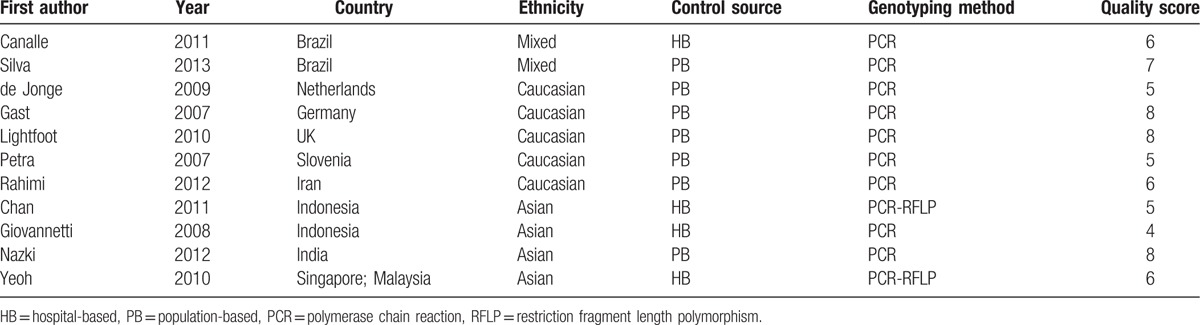
Main features of eligible investigations for meta-analysis.

**Table 2:**
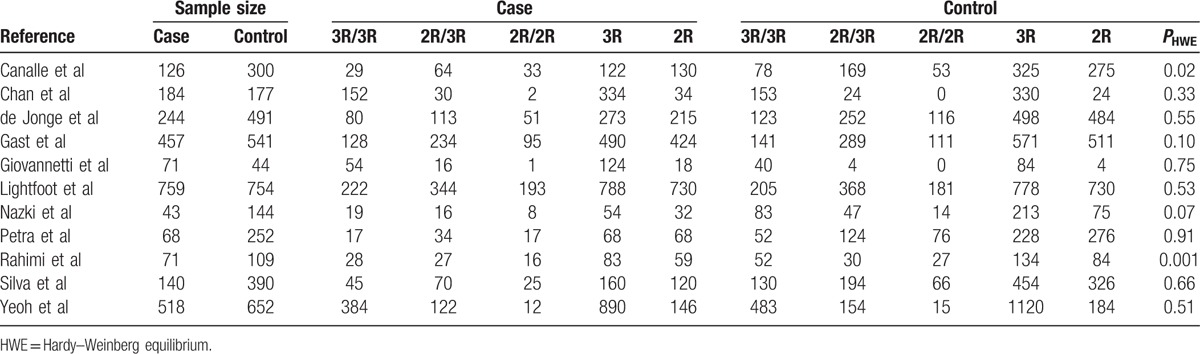
Genotypes distribution data of TSER variation among cases and controls.

### 3.2 Quantitative synthesis results

**Figure 2:**
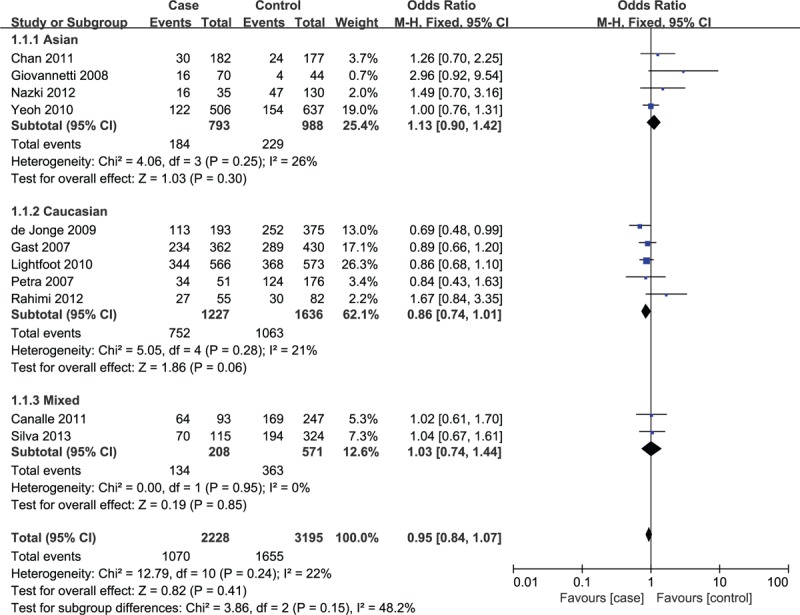
Forest plot about association of TSER variation and pediatric ALL risk under the heterozygote model (2R/3R vs 3R/3R). ALL = acute lymphoblastic leukemia, CI = confidence interval, M–H = Mantel–Haenszel method, TSER = thymidylate synthase enhancer region.

The main results of heterogeneity estimate and quantitative synthesis are summarized in Table 3. No significant heterogeneity was detected between the included investigations in all 5 genetic models for TSER polymorphism. If all the eligible investigations were combined into the quantitative analysis, the results found no statistically significant association between TSER variation and susceptibility to pediatric ALL under 5 genetic models (2R/3R vs 3R/3R: OR = 0.95, 95% CI = 0.84–1.07, *P* = 0.41; 2R/2R vs 3R/3R: OR = 0.99, 95% CI = 0.84–1.16, *P* = 0.90; 2R2R vs 3R/3R+2R/3R: OR = 1.05, 95% CI = 0.92–1.21, *P* = 0.45; 2R/3R+2R/2R vs 3R/3R: OR = 0.97, 95% CI = 0.87–1.09, *P* = 0.63; 2R vs 3R: OR = 1.03, 95% CI = 0.92–1.15, *P* = 0.61). Similarly, no significant association was found in the stratification analyses according to ethnicity (Asian, Caucasian, and Mixed), NOS score (low quality and high quality), and control source (hospital-based and population-based) (Fig. 2, Table 3).

**Table 3:**
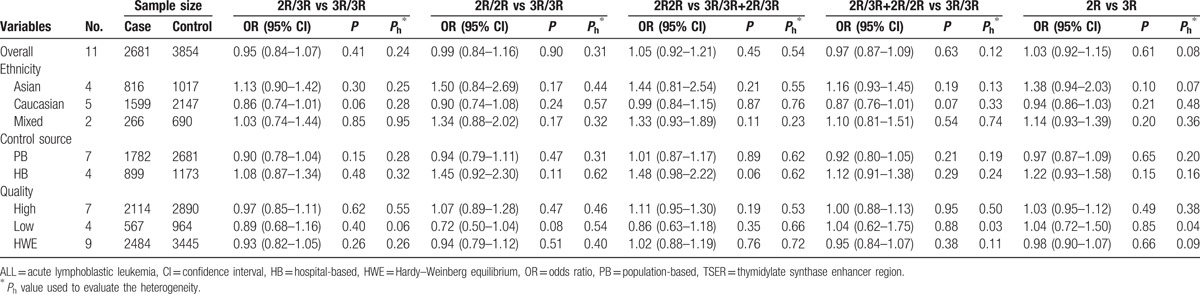
Results of quantitative analysis for TSER variation and pediatric ALL risk.

### 3.3 Publication bias and sensitivity analysis

**Figure 3:**
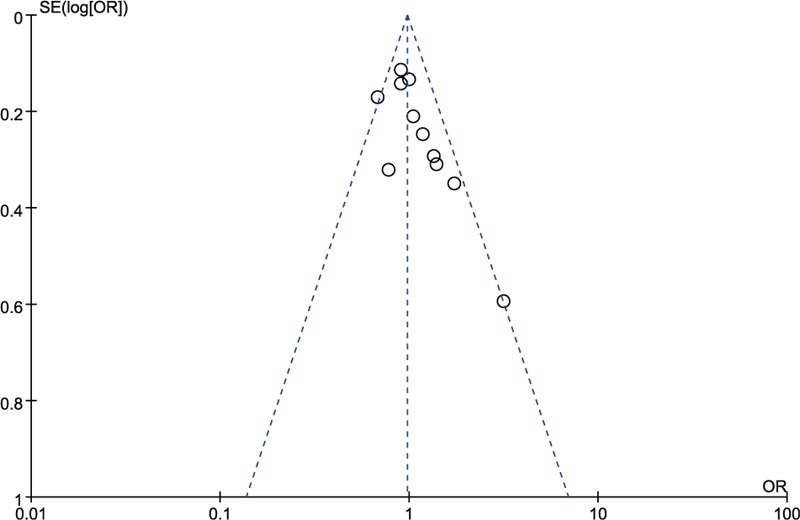
Funnel plot assessing publication bias in dominant model (2R/3R+2R/2R vs 3R/3R). OR = odds ratio, SE = standard error.

Sensitivity analysis, in which the pooled ORs were recalculated after removal investigations not in consistent with HWE, revealed that the combined results remained virtually unchanged, suggesting the robustness of our results (Table 3). The shapes of inverted funnel plots were symmetrical, which suggested that no obvious publication bias was found (Fig. 3). In addition, the results of Egger test also had no statistical significance for the assessment of publication bias.

## 4 Discussion

TYMS, a key enzyme participated in the DNA biosynthesis, catalyzes the conversion of dUMP to dTMP to provide the only de novo synthesis of thymidine.^[34]^ It has been proved that TYMS regulates the expression of some crucial cancer genes as an RNA-binding protein via translational repression.^[35–37]^ Therefore, alteration in TYMS activity is thought to be connected with tumorigenesis through disruption of genome integrity, imbalance in repair mechanisms, changes of methylation status, and cell cycle dysregulation. Moreover, TYMS is one of the therapeutic targets for many chemotherapeutic drugs like methotrexate and 5-fluorouracil.^[38]^ The described several functional variants of *TYMS* gene untranslated regions affect *TYMS* mRNA stability, transcription, or protein expression. It has been reported that the 3R form of TSER variation was related to a higher transcription level of *TYMS* than those with 2R form.^[17,39]^ It is plausible to speculate that TSER polymorphism might lead to alterations in DNA biosynthesis and methylation, and influence the cancer susceptibility.

To date, many epidemiological studies regarding the association of TSER variation with risk of pediatric ALL have been reported, but the published results remain controversial. Gast et al^[26]^ found no statistical differences in genotype and allele distribution for TSER polymorphism between children with ALL and the controls. Canalle et al^[23]^ showed that, compared with children who carried only 2R form, individuals who carried 3R form of TSER had a significantly reduced risk to develop pediatric ALL. Reduced leukemia risk was also observed for the 3R2R variant (OR = 0.7, 95% CI = 0.4–1.0, *P* = 0.04) and 2R allelic carriers (OR = 0.7, 95% CI = 0.5–1.0, *P* = 0.03) in de Jonge et al study.^[25]^ To elucidate this inconsistency, a synthetical meta-analysis was conducted. In our study, no significant heterogeneity was observed among overall studies under all 5 genetic models. The combined data demonstrated that there was no significant association of TSER variation and risk of pediatric ALL in overall comparison under all genetic models. No significant association was found in the stratification analyses based on ethnicity, control source, and quality score. Our results were not in accordance with the conclusion published by Weng et al,^[40]^ which showed TSER variation might dedicate to significantly increased risk of childhood ALL (3R/3R vs 2R/2R: OR = 1.46, 95% CI = 1.03–2.06). Since our study added several new investigations and included 2681 children with ALL and 3854 matched controls, which allowed for sufficient statistical power and more precise estimation, our conclusion is more reliable.

However, several limitations in our study need to be addressed in interpreting the results. First, due to data insufficiency, 2 relevant investigations were removed from the quantitative synthesis. Second, our analysis largely focused on single-factor estimates not adjusted for other confounders such as gender, lifestyles, and other potential factors, which may cause confounding bias and influence the combined results. The combined analyses of some subgroups may have no sufficient testing power to accurately assess the real association. In addition, the gene-environment interactions that may modify cancer susceptibility were not assessed in our study ascribed to the limited relevant information.

## 5 Conclusion

In brief, this meta-analysis suggested that TSER polymorphism in *TYMS* gene was not related to susceptibility to develop pediatric ALL. However, in the future, well-designed studies with more participants are demanded to verify this conclusion.

## Supplementary Material

Supplemental Digital Content
