# Supplementary material for: TSER polymorphism is not associated with risk of pediatric acute lymphoblastic leukemia: A meta-analysis
Source: Medicine (Baltimore). 2017 Feb 17;96(7):e6143. doi: 10.1097/MD.0000000000006143 (PMC5319533; doi:10.1097/MD.0000000000006143)

**Supplemental Figure**. Overview of the folate metabolic pathway.

DHF = dihydrofolate, DHFR = dihydrofolate reductase, dTMP = deoxythymidine monophosphate, dUMP = deoxyuridine monophosphate, MTHFR = 5,10-methylenetetrahydrofolate reductase, MTR = methionine synthase, MTRR = methionine synthase reductase, SAH = S-adenosylhomocysteine, SAM = S-adenosylmethionine, SHMT = serine hydroxymethyltransferase, THF = tetrahydrofolate, TYMS = thymidylate synthase


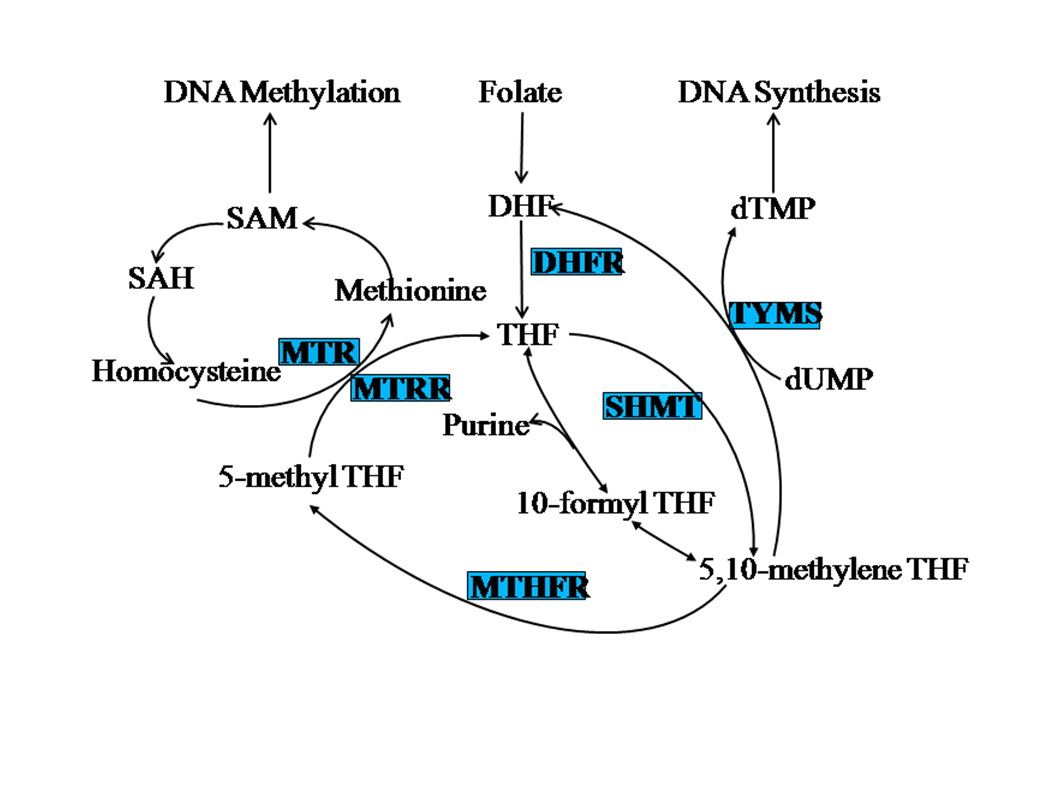

Supplement: Supplemental Digital Content [file medi-96-e6143-s001.doc]
